# Head Impact Exposure in Youth Football

**DOI:** 10.1007/s10439-012-0530-7

**Published:** 2012-02-15

**Authors:** Ray W. Daniel, Steven Rowson, Stefan M. Duma

**Affiliations:** Center for Injury Biomechanics, Virginia Tech-Wake Forest University, 440 ICTAS Building, Stanger St., Blacksburg, VA 24061 USA

**Keywords:** Concussion, Brain injury, Biomechanics, Helmet, Linear, Rotational, Acceleration, Pediatric, Children

## Abstract

The head impact exposure for athletes involved in football at the college and high school levels has been well documented; however, the head impact exposure of the youth population involved with football has yet to be investigated, despite its dramatically larger population. The objective of this study was to investigate the head impact exposure in youth football. Impacts were monitored using a custom 12 accelerometer array equipped inside the helmets of seven players aged 7–8 years old during each game and practice for an entire season. A total of 748 impacts were collected from the 7 participating players during the season, with an average of 107 impacts per player. Linear accelerations ranged from 10 to 100 g, and the rotational accelerations ranged from 52 to 7694 rad/s^2^. The majority of the high level impacts occurred during practices, with 29 of the 38 impacts above 40 g occurring in practices. Although less frequent, youth football can produce high head accelerations in the range of concussion causing impacts measured in adults. In order to minimize these most severe head impacts, youth football practices should be modified to eliminate high impact drills that do not replicate the game situations.

## Introduction

Sports related concussions have received increased public awareness, with many states considering or implementing laws directing the response to suspected brain injury. This is a result new research suggesting possible links to long-term consequences from repetitive concussions.^13^,^21^,^22^ Emergency department visits for concussions increased 62% between 2001 and 2009, and researchers estimate that between 1.6 and 3.8 million sports related concussion occur each year in the United States.^5^,^19^ Of all sports, football accounts for the highest incidence of concussion, and therefore receives the most attention.^34^ One of the leading thoughts to minimize the incidence of concussion in football is to limit players’ exposure to head impacts.^9^ Strategies to reduce a player’s exposure to head impact include teaching proper tackling techniques and modifying the rules of the game.

To make educated decisions toward reducing the incidence of concussion in football, head impacts in football have been extensively studied over the past decade.^2^,^8^,^10^–^12^,^15^,^16^,^20^,^23^,^26^,^30^ The National Football League (NFL) was the first to investigate this problem in detail by reconstructing concussive impacts through analysis of game film using instrumented crash test dummies.^23^–^26^ While this work was of high quality, it was limited by a dataset that did not account for the full exposure to head impacts that players experienced.^30^,^32^ Since then, new technology, the Head Impact Telemetry (HIT) System (Simbex, Lebanon, NH), has allowed for the direct instrumentation of headgear in sports.^7^,^14^,^18^,^28^ The HIT System consists of a series of accelerometers that fit inside football helmets, and records a player’s biomechanical head response to every head impact they receive. Since Virginia Tech first instrumented college football players with the HIT System in 2003, over 1.5 million head impacts have been collected and analyzed across participating institutions.^12^ This has allowed head impact exposure and injury risk to be investigated at the high school and college level.^1^,^2^,^4^,^8^,^10^,^11^,^15^,^16^,^20^,^29^,^30^,^32^,^33^ Based on this research, some colleges have made educated recommendations about contact in practices in an effort to reduce the head impact exposure of players. Furthermore, this research has led to design guidelines for improved adult football helmets.^30^

There are approximately 5 million athletes participating in organized football in the United States; with 2000 NFL players, 100,000 college players, 1.3 million high school players, and 3.5 million youth players.^17^,^27^ Previous research has investigated head impacts in high school football, college football, and the NFL; however, this population only accounts for 30% of football players. To date, no work has been performed investigating head impact exposure in youth football, which accounts for 70% of all football players. Investigating head impact exposure at the youth level would allow researchers to understand when head impacts occur most frequently and which activities cause the most severe impacts. With this increased understanding, educated decisions can be made to effectively minimize head impact exposure in youth football.

The objective of this study was to investigate the head impact exposure in youth football. This was accomplished by instrumenting the helmets of a youth football team with head acceleration measurement devices similar to the HIT System. Youth head impact data are reported and compared to that of the high school and college levels of play. These data are the first step toward educated decisions about changes to youth football, and have applications toward youth-specific football helmet designs.

## Materials and Methods

A youth football team consisting of children ranging in age from 6 to 9 years old participated in this study approved by the Virginia Tech Institutional Review Board. Each player gave assent and their parental guardians provided written informed permission. This study investigated head impact exposure in youth football by instrumenting the helmets of youth football players with a custom six degree of freedom (6DOF) head acceleration measurement device.^28^,^29^ Of the 26 players on the youth team, the helmets of seven players were instrumented with the 6DOF measurement device. The seven players had an average body mass 31.7 ± 6.44 kg and were all 7 or 8 years old. The players were chosen due to anticipation of high participation in practices and games, as well as playing both offense and defense. Furthermore, these players wore youth medium or youth large sized Riddell Revolution (Elyria, OH) helmets that were compatible with the 6DOF measurement device.

**Figure 1:**
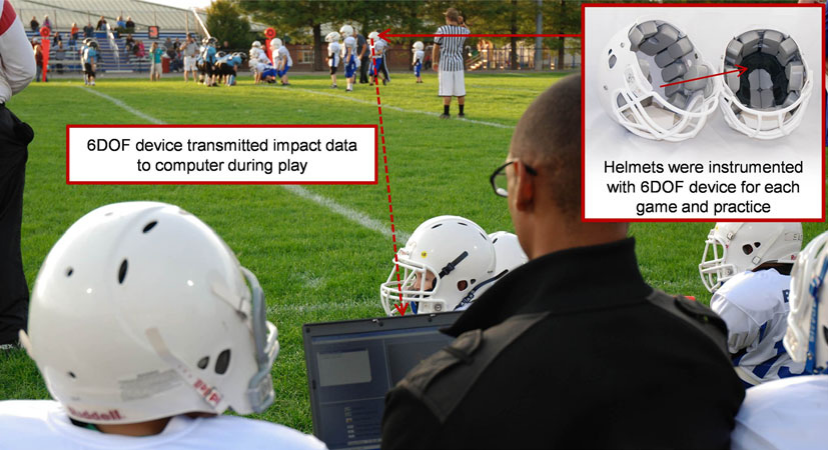
The helmets of youth football players were instrumented with the 6DOF head acceleration measurement device. Players wore instrumented helmets for every game and practice they participated in. Each time an instrumented player experienced a head impact, data were collected and then wirelessly transmitted to a computer on the sideline

The 6DOF measurement device consists of 12 accelerometers and is designed to integrate into Riddell Revolution football helmets (Fig. 1). While the 6DOF measurement device was originally designed for adult Revolution football helmets, the device is compatible with youth helmets due to the same sizing conventions and identical padding geometries between adult and youth Revolution helmets. Instrumented helmets were worn by youth football players during each game and practice they participated in. Each time an instrumented helmet was impacted and an accelerometer exceeded a specified threshold, data acquisition was automatically triggered. A total of 40 ms of data from each accelerometer were recorded, including 8 ms of pre-trigger data. Once data acquisition was complete, data were wirelessly transmitted to a computer on the sideline. Acceleration data were then processed to compute linear and rotational head acceleration using a novel algorithm.^6^,^28^ While a brief overview of the 6DOF measurement device is presented here, a detailed technical description has previously been reported.^28^

Impact location for each head impact recorded was determined from the acceleration traces using methods that have been previously described.^14^ All head impacts were generalized into one of four impact locations on the helmet: front, side, rear, and top. Overall acceleration distributions were analyzed by impact location. Overall accelerations distributions were also analyzed by session type, which was divided into practices and games. Head impact exposure is presented in terms of the frequency of impacts, median accelerations, and 95th percentile accelerations. Furthermore, empirical cumulative distribution functions (CDF) with 95th percentile confidence intervals were computed for linear and rotational acceleration. Results of this study are then compared to studies quantifying head impact exposure in high school and college football players.

## Results

**Figure 2:**
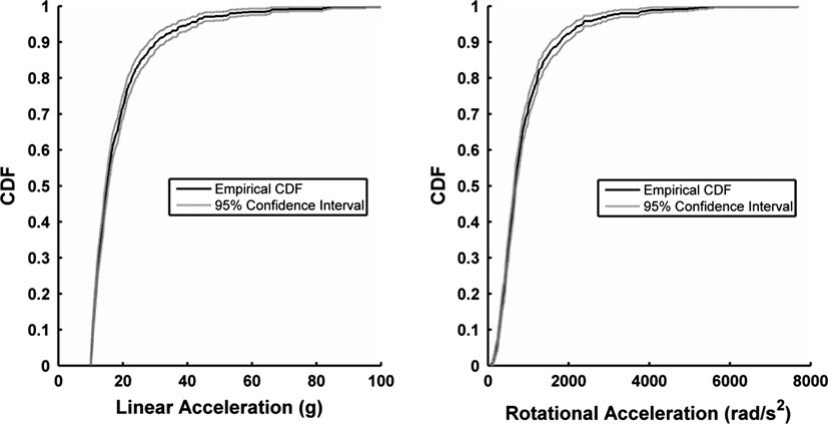
Cumulative distribution functions for linear and rotational accelerations show that the distribution of impacts were right skewed and heavily weighted toward low magnitude impacts

Both the linear and rotational acceleration distributions were right-skewed, and heavily weighted toward low magnitude impacts. CDF for resultant linear and rotational accelerations with 95th percentile confidence intervals were determined (Fig. 2). Linear accelerations ranged from 10 to 100 g. The distribution of linear acceleration had an average value of 18 g, a median value of 15 g, and a 95th percentile value of 40 g. Rotational accelerations ranged from 52 to 7694 rad/s^2^. The distribution of rotational acceleration had an average value of 901 rad/s^2^, a median value of 671 rad/s^2^, and a 95th percentile value of 2347 rad/s^2^.

A total of 748 impacts were recorded during practices and games for the seven instrumented players during the youth football season. During games, 307 impacts (41% of total) were collected, while 441 impacts (59% of total) were collected during practices. The average instrumented player experienced at least one impact greater than 10 g in 14.1 sessions, consisting of 4.7 games and 9.4 practices. The average instrumented player experienced 107 head impacts, which included 44 impacts during games and 63 impacts during practices. Furthermore, the average player experienced 6.7 impacts per practice and 5.8 impacts per game. A total of 38 impacts above 40 g were collected, 29 of which occurred during practices. A total of 6 impacts were collected with linear accelerations above 80 g, with all six occurring in practices. No instrumented players sustained a concussion throughout the season.

Impacts to the sides of the helmet were most common, accounting for 36% of all impacts. The front of the helmet received approximately 31% of all the impacts. The top and rear of the helmet were impacted least frequently, accounting for 18 and 14% of all impacts, respectively. Impacts to the top of the helmet exhibited the greatest magnitudes of linear acceleration, while impacts to the sides of the helmet resulted in the greatest magnitudes of rotational acceleration (Table 1).

**Table 1:** Comparison of head impact exposure across impact locations

Impact location	Number of impacts	Linear acceleration (g)		Rotational acceleration (rad/s^2^)	
		Median (50%)	95%	Median (50%)	95%
Front	235	14	28	670	1516
Side	272	14	25	747	2104
Rear	106	15	30	679	2057
Top	135	20	45	467	1483

Impacts to the side of the helmet were most frequent and resulted in the greatest rotational accelerations. Impacts to the top of the helmet were less frequent, but resulted in the greatest linear accelerations

## Discussion

This study reports, for the first time, the head impact biomechanics experienced with participation in youth football. From these data, how frequently and how severely 7 and 8 year old children impact their heads while playing in organized tackle football can be characterized. Interestingly, high magnitude impacts (>80 g) were experienced by the instrumented children during play. This level of severity is similar to some of the more severe impacts that college players experience, even though the youth players have less body mass and play at slower speeds.^30^ These data serve as the basis of educated decisions related to rule changes and practice structure in youth football, as well as design criteria for youth-specific football helmets.

Of the 107 head impacts the average player sustained, 59% occurred during practices and 41% occurred during games. This was not solely attributed to the average player participating in more practices than games (9.4 practices to 4.7 games), as players experienced 15% more impacts per practice than per game. More notably, impacts of higher magnitude were associated with practices rather than games, where 76% of impacts greater than 40 g and 100% of impacts greater than 80 g occurred during practices. This contrasts trends exhibited in high school and college football, where more severe impacts are associated with games.^2^,^8^,^10^,^33^ Head impact exposure in youth football, particularly at higher severities, can be reduced through evaluating and restructuring practices. This can be achieved through teaching proper tackling techniques and minimizing drills that involve full contact; and instead, focusing on practicing fundamental skill sets needed in football at these young ages.

Head impact exposure in football has two components: frequency of impacts and magnitude of impacts. While this study is the first to report on head impact exposure in youth football, research quantifying head impact exposure in high school and college football has been ongoing for the last decade.^12^ When comparing the frequency component of head impact exposure across level of play, the number of head impacts a player sustains each season rises with increasing level of play (Table 2). This is not unexpected, as the youth football season (in terms of the number of practices and games, as well as session length) is shorter than the high school football season, which is shorter than the college football season. When comparing the magnitude component of head impact exposure across level of play, the 95th percentile impact increases with level of play for both linear and rotational acceleration, which is indicative of how frequently high magnitude impacts are sustained by players (Table 2). This finding is also not surprising, as the size of the players and speed of play both increase with age. With that said, it is important to note that all levels of play experience high magnitude impacts (>80 g), but these impacts occur more frequently as the player gets older.

**Table 2:** Comparison of head impact exposure between youth, high school, and college football

Level of play	Impacts per season	Linear acceleration (g)		Rotational acceleration (rad/s^2^)	
		Median (50%)	95%	Median (50%)	95%
Youth (7–8 years)	107	15	40	672	2347
High school (14–18 years)	565	21	56	903	2527
College (19–23 years)	1000	18	63	981	2975

The number of impacts per season and distribution of magnitudes both increase as the players get older. These data were quantified from studies using similar methodologies to instrument youth, high school, and college football players^1^,^3^,^30^,^31^

**Figure 3:**
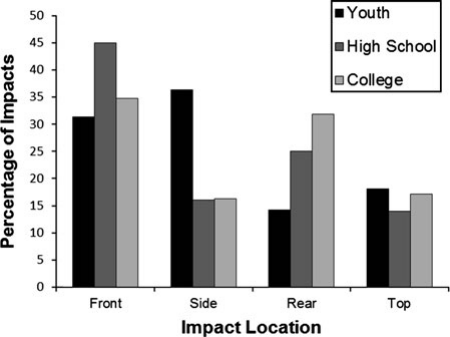
Comparison of helmet impact location distributions between youth, high school, and college football. Youth players impact the side of the helmets more and rear of their helmets less than high school and college players

The head impact data can be further analyzed by the distribution of helmet impact locations. The instrumented youth players impacted the side of their helmets most frequently. When compared to high school and college impact distributions, youth players experienced a substantially higher percentage of impacts to the side of the helmet and a substantially lower percentage of impacts to the rear of the helmet (Fig. 3). This can likely be attributed to the differences in the style of play between the different age groups, as well as the youth players having a tendency to fall to the side while being tackled. Furthermore, the helmets that the youth players wear may influence some of these trends. Youth football helmets are very similar in size and mass to adult football helmets. With that said, the neck muscles of 7–8 year olds are undeveloped in comparison to high school and college football players. These two factors may result in a youth player being more susceptible to impacting his head on the ground while being tackled than a high school or college player.

Moreover, these data have applications toward future youth helmet design. Currently, youth football helmets are remarkably similar to adult helmets in relation to size, mass, and design materials. In the past, researchers have used data collected from instrumented college football players to develop the STAR evaluation system that assesses a helmet’s overall ability to reduce the probability of concussion.^30^ This evaluation system is derived from quantified head impact exposure in college football. Head impact exposure measured on the field is related to laboratory tests that evaluate impact performance. The results of the laboratory tests are then disseminated to the public to provide information to consumers on relative helmet performance. Furthermore, the STAR evaluation system provides manufacturers with design guidelines to improve future helmet safety. Unfortunately, this system cannot be extrapolated to youth football helmets because the head impact exposure of youth football is different than that of college football. This study is an important step toward development of a helmet evaluation system for youth football, which would provide guidelines for designing youth-specific football helmets. While this study provides a first glimpse of head impact exposure in youth football, more data is currently needed across the age continuum (6–13 years old) of youth football.

This study has several limitations. First, it should be noted that a total of seven youth football players were included in this study. This is a small sample size in comparison to some of the studies investigating head impact exposure in high school (95 players) and college (>300 players) football.^4^,^32^ Second, the instrumented players ranged in age from 7 to 8 years old. However, youth football encompasses players ranging in age from 6 to 13 years old. A larger sample size of players ranging from 6 to 13 years old is needed to completely define head impact exposure in youth football. Third, the 6DOF measurement device is associated with some measurement error. However, average acceleration measurement error is on the order of 1–3%.^28^ While there may be greater error associated with individual data points, these errors are of little consequence when working with the overall data distributions.

In conclusion, this study is the first to report the head impact biomechanics associated with youth football. Valuable insight to the head impact exposure in youth football has been presented. While youth football players impact their heads less frequently than high school and college players, and have impact distributions more heavily weighted toward low magnitude impacts; high magnitude impacts still occur. Interestingly, the majority of these high magnitude impacts occur during practice. Restructuring youth football practices may be an effective method of reducing the head impact exposure in youth football. These data are the basis of educated decisions about future changes to youth football and have applications toward determining guidelines for youth-specific helmet design.
